# Networked Sensor-Based Adaptive Traffic Signal Control for Dynamic Flow Optimization

**DOI:** 10.3390/s25113501

**Published:** 2025-06-01

**Authors:** Xinhai Wang, Wenhua Shao

**Affiliations:** School of Computer Science, Beijing University of Posts and Telecommunications, Beijing 100876, China; kelly@bupt.edu.cn

**Keywords:** sensor networks, traffic flow, traffic signal timing, adaptive control, PID controller

## Abstract

With the rapid advancement of modern society, the demand for efficient and convenient transportation has increased significantly, making traffic congestion a pressing challenge that must be addressed in the process of urban expansion. To effectively mitigate this issue, we propose an approach that leverages sensor networks to monitor real-time traffic data across road networks, enabling the precise characterization of traffic flow dynamics. This method integrates the Webster algorithm with a proportional–integral–derivative (PID) controller, whose parameters are optimized using a genetic algorithm, thereby facilitating scientifically informed traffic signal timing strategies for enhanced traffic regulation. Geomagnetic sensors are deployed along the roads at a ratio of 1:50–1:60, and radar sensors are deployed on the roadsides of key sections. This can effectively detect changes in road traffic flow and provide early warnings for possible accidents. The integration of the Webster method with a genetically optimized PID controller enables adaptive traffic signal timing with minimal energy consumption, effectively reducing road occupancy rates and mitigating congestion-related risks. Compared to conventional fixed-time control schemes, the proposed approach improves traffic regulation efficiency by 17.3%. Furthermore, it surpasses traditional real-time adaptive control strategies by 3% while significantly lowering communication energy expenditure. Notably, during peak hours, the genetically optimized PID controller enhances traffic control effectiveness by 13% relative to its non-optimized counterpart. A framework is proposed to improve the efficiency of road operation under the condition of random traffic changes. The k-means method is used to mark key roads, and weights are assigned based on this to coordinate and regulate traffic conditions. These findings underscore our contribution to the field of intelligent transportation systems by presenting a novel, energy-efficient, and highly effective traffic management solution. The proposed method not only advances the scientific understanding of dynamic traffic control but also offers a robust technical foundation for alleviating urban traffic congestion and improving overall travel efficiency.

## 1. Introduction

In recent decades, the global trend of rapid urbanization has been accompanied by a substantial surge in the number of motor vehicles and licensed drivers. In China alone, by the end of 2024, the motor vehicle fleet had swelled to an impressive 453 million, while the number of licensed drivers exceeded 542 million. These figures reflect an annual growth rate of 10–15%, indicating a continuous and significant expansion of the automotive landscape [1,2].

This exponential growth in vehicle ownership has led to a plethora of urban transportation challenges. A growing number of regions are now grappling with the reality that peak-hour traffic demand far exceeds the existing service capacity of their transportation infrastructure. As a result, urban congestion has become an increasingly pressing issue, with 44% of Chinese cities experiencing suboptimal traffic conditions in 2024. This phenomenon is not unique to China; it is a global concern that has garnered attention from international bodies and conferences. For instance, the 2021 United Nations Sustainable Transport Conference and the 2023 World Intelligent Transport Congress both emphasized the urgent need for the development of intelligent and sustainable transportation systems to support the sustainable growth of urban areas.

The urban road network is primarily composed of road segments and intersections, which are the two key components where traffic congestion often originates. On the one hand, accidents occurring on road segments can disrupt the normal flow of traffic, leading to bottlenecks and delays. On the other hand, intersections are critical points where traffic from multiple directions converges, and the efficiency of these intersections is heavily dependent on the alignment of traffic demand with the implemented traffic control strategies. When there is a mismatch between the control strategies and the actual traffic demand, congestion can be significantly exacerbated [3,4].

To address the growing problem of traffic congestion and enhance the overall operational efficiency of urban areas, it is essential to implement a two-pronged approach. Firstly, early warning systems need to be established for high-risk road segments that experience heavy traffic flow. These systems can help identify potential congestion points in advance, allowing for proactive measures to be taken to mitigate the impact of traffic buildup. Secondly, the service capacity of intersections needs to be enhanced to better adapt to the dynamic nature of traffic demand. This can be achieved through the implementation of optimized traffic signal control strategies that aim to reduce road occupancy, curb queue lengths, and minimize waiting times [5,6].

The performance of traffic signal control strategies can be evaluated based on various metrics, such as segment occupancy rates over defined time intervals. Among the existing traffic control methodologies, the Webster approach is widely recognized as one of the most traditional and fundamental methods. However, it has been observed that when there are significant disparities in traffic flow between different road segments, the Webster method may lead to an inequitable allocation of resources, particularly favoring high-flow segments at the expense of low-flow segments [7].

To overcome the limitations of the Webster method, several researchers have proposed enhancements. For example, Guo et al. [8] integrated a PID controller into the Webster method, aiming to improve its performance. However, this enhanced approach still has some drawbacks. It requires manual parameter tuning, which can be a time-consuming and labor-intensive process. Moreover, it lacks the capability for autonomous adaptation to fluctuating traffic conditions, making it less effective in dynamic traffic environments.

In addition to the Webster–PID controller approach, adaptive traffic signal control methods based on reinforcement learning have also been explored in the literature [7]. While these methods have shown promise in terms of their ability to adapt to changing traffic conditions, they impose substantial real-time communication demands and energy consumption, which can be a significant barrier to their widespread implementation.

In light of the limitations of the existing traffic signal control methodologies, this study delves deeper into the field of traffic signal control. It focuses on refining the Webster–PID controller approach and juxtaposing it against adaptive algorithms to evaluate their relative performance and effectiveness. This study’s contributions are manifold and are expected to provide valuable insights into the development of more efficient and sustainable traffic signal control strategies for urban areas.

(1) A hybrid sensor network is deployed within SUMO simulations to monitor road occupancy rates and issue early warnings for potential congestion and accident risks.
(2) An enhanced Webster–PID algorithm is proposed, and the genetic algorithm is used to optimize the effect of the PID controller. It is verified that the control effect can be effectively enhanced when the genetic algorithm is used to uniformly tune the PID controller parameters.
(3) In a complex road network, k-means is used to determine the intersections with greater influence, and the dynamic weight of each intersection during regulation is obtained. The traffic light timing scheme is adjusted to achieve real-time optimization of road conditions.

The remainder of this paper is organized as follows: Section 2 presents the related works and the optimization method of the algorithm. Section 3 explains the modeling method and the simulation results. The analysis is discussed in Section 4. Finally, the conclusions are presented in Section 5.

## 2. Materials and Methods

### 2.1. Related Works

In the domain of traffic flow detection utilizing sensor networks, extensive research has been conducted both domestically and internationally, yielding significant advancements.

Among these studies, Sing Yiu Cheung et al. [9] demonstrated that wireless sensors can achieve a traffic flow detection accuracy of up to 99%, surpassing other sensor types in both precision and cost-effectiveness, thereby validating the feasibility of wireless sensors for traffic monitoring applications. Wenjie Chen et al. [10] further developed a prototype wireless sensor network for real-time data collection and transmission, employing three types of Wireless Intelligent Transportation System (WITS) nodes for traffic detection. In China, although research on sensor network systems began relatively late, it has garnered increasing attention in recent years. The national 10th Five-Year Plan identified sensor networks as a key strategic area for scientific and technological advancement. Tian Yin et al. [11] constructed an urban traffic sensing system with a universal architecture, integrating intelligent sensing technology into urban traffic management. By establishing a sensor network, they proposed a methodology for the accurate detection of traffic flow and vehicle classification.

These studies collectively highlight the critical role of sensor networks in monitoring road conditions and facilitating data-driven traffic regulation.

Similarly, substantial progress has been achieved in traffic signal control optimization. Juan Moreno-Malo et al. [12] introduced a multi-agent framework for traffic signal control, significantly reducing vehicle waiting times compared to conventional timing schemes. Liu Junxiu et al. [13] refined the reward function in reinforcement learning, proposing an adaptive traffic control system capable of maintaining robust performance even in complex and dynamic traffic environments. Cai Jinde et al. [14] integrated queue length considerations into a traffic signal timing optimization model, utilizing the Analytic Hierarchy Process (AHP) to determine weighting coefficients and employing a fixed-step search method to enhance the dynamic regulation of traffic signals. Shou Yanfang et al. [15] developed an optimization model that accounts for both traffic delay and exhaust emissions, refining signal control through the optimization of cycle length, green split, and phase sequencing while introducing a satisfaction evaluation function to improve overall signal timing assessment. In recent years, many scholars have also made improvements to traffic light control by using methods such as reinforcement learning. Mohamad Belal Natafgi et al. [16] incorporated reinforcement learning into adaptive traffic signal control systems, effectively decreasing both average queue lengths and waiting times. Lin, Zhongjie, et al. [17] improved three well-known meta-heuristic algorithms, namely, the genetic algorithm (GA), particle swarm optimization algorithm (PSO), and differential evolution algorithm (DE), to solve the multi-objective urban traffic light scheduling problem. Z. Lin et al. [18] proposed a multi-agent adaptive generalized DRL (ABDRL) method for traffic light control (TLC) that combines generalized networks with deep network structures to improve the optimality and robustness of traffic light scheduling schemes.

However, existing approaches exhibit certain limitations. While previous research has examined the impact of sensor network layouts on data accuracy and the effectiveness of traffic signal adjustments based on collected data, it has not directly addressed the influence of factors such as sensor network deployment density and data sampling frequency on traffic signal optimization.

Additionally, some studies have recognized the necessity of integrating traffic detection systems with signal control strategies. For example, Chabchoub et al. [19] designed an intelligent traffic light control system that combines fuzzy logic with MATLAB-based image processing techniques to alleviate urban congestion. Amnesh Goel [20] proposed a wireless sensor network-based adaptive intersection system designed to prioritize emergency and special-purpose vehicles.

Thus, there is a compelling need to explore more precise and reliable methodologies beyond conventional adaptive control strategies. By leveraging sensor network data for the real-time optimization of traffic signal timing, urban traffic management efficiency can be significantly improved. To address these challenges, we conducted an in-depth analysis of the impact of sensor network characteristics on data acquisition and traffic signal timing optimization.

Based on this analysis, we developed an adaptive coordinated control strategy for multi-intersection intelligent traffic signals, enabling dynamic, real-time, and data-driven regulation. This approach replaces conventional rule-based control schemes with more advanced, data-driven methodologies. Its effectiveness is validated through simulation experiments, providing both theoretical foundations and practical guidance for enhancing urban traffic management.

### 2.2. Methodology

#### 2.2.1. Sensor Network Construction

The present section provides a theoretical exposition of the key methodologies employed in this study, centering on the deployment of roadway sensor networks and the algorithmic frameworks implemented for adaptive traffic signal control.

Table 1 shows the relevant costs of deploying different sensors, and Table 2 shows the accuracy of different sensors in perceiving road conditions. According to comprehensive statistical analyses and comparative evaluations of sensor performance under varying traffic conditions [21], embedded sensors such as inductive loop detectors and geomagnetic sensors have demonstrated vehicle counting accuracy rates of 99% or higher. Among these, inductive loops, geomagnetic sensors, and microwave radar sensors exhibit consistent and reliable performance across diverse traffic flow scenarios.

When the magnetic field on the bridge circuit changes, it causes the resistance value in the corresponding direction to either increase or decrease. The resulting output voltage, which can indicate the presence of vehicles within the sensor’s detection range [22,23,24], can be calculated using Equation (1):

(1) $$V_{\mathrm{out}}=\frac{V_{cc}}{2R}(R+dR)-\frac{V_{cc}}{2R}(R-dR)=\frac{dR}{R}V_{cc},$$

Given the differences in detection range, operational characteristics, and cost among various sensor types, we propose a hybrid sensor network architecture that strategically integrates multiple sensing technologies. Specifically, geomagnetic sensors are deployed across all road segments for broad-area coverage [25,26], while radar sensors are installed at critical monitoring points to enhance detection precision. This dual-sensor network configuration facilitates the cross-validation of collected traffic data, enabling error identification and correction through data fusion. Compared to single-sensor systems, this integrated approach significantly enhances the comprehensiveness of road condition monitoring by leveraging complementary detection mechanisms, ultimately improving the accuracy and reliability of real-time traffic flow analysis.

#### 2.2.2. Optimize the Results of the Webster Method in Combination with PID Controller

The traffic data acquired through geomagnetic sensors and other monitoring devices serve as a fundamental basis for optimizing traffic signal durations. Currently, the primary methodologies for traffic signal timing adjustment include fixed-time control, fuzzy rule-based control [27,28], simple adaptive control [29,30,31,32,33,34], the classical Webster method [35,36,37,38], and the methods based on Q learning [39].

Among these, fixed-time control is based on the long-term statistical analysis of traffic flow at specific road sections. By continuously monitoring intersection traffic conditions, the flow ratio for each approach is calculated, allowing for the design of a predetermined signal-timing scheme [40]. While this method ensures stability and imposes minimal requirements on signal transmission infrastructure, it lacks responsiveness to sudden fluctuations in traffic flow, often leading to suboptimal control performance.

The Webster method remains a widely utilized classical approach for determining preliminary signal cycle durations based on traffic flow and lane capacity. Its core principle lies in the computation of an optimal cycle length to balance efficiency and throughput. However, this method relies entirely on traffic flow calculations, which introduces certain limitations. Specifically, when traffic flow is too low, the computed cycle duration may be excessively short, increasing the likelihood of unnecessary stops and traffic accidents. Conversely, under high traffic volumes, the method may disproportionately allocate green time to heavily congested approaches, exacerbating delays for other directions. As a result, additional constraints, such as minimum cycle lengths and adaptive cycle adjustments, are often required to refine the model.

The key computational steps of the Webster method are as follows:

1. Determine the lane saturation flow rate for each intersection.
2. Calculate the ratio of the actual flow rate to the saturation flow rate for each critical lane Y.
3. Estimate the total lost time.
4. Compute the total cycle time: Integrating the above parameters, the total cycle length is derived using Equation (2), ensuring an optimal balance between traffic demand and signal timing efficiency.

By refining these calculations and incorporating real-time traffic data, the Webster method can be further enhanced to improve adaptability and operational performance in dynamic urban traffic environments.

(2) $$C=\frac{1.5L+5}{1-Y},$$

Among them, C represents the signal cycle (including both red and green light durations), L denotes the lost time, and Y indicates the traffic flow ratio.

The final green time duration $G_{e}$ is calculated using Equation (3).

(3) $$G_{e}=(C-L)\frac{y_{i}}{Y},$$

After implementing basic adjustments to the traffic signal timing, further refinements are required based on specific conditions. Therefore, an appropriate method must be introduced in subsequent stages to perform more precise adjustments according to real-time traffic flow conditions.

For the system comprising roadways and traffic flows, traffic signal control constitutes a closed-loop feedback mechanism. Such closed-loop feedback control strategies include fuzzy feedback, adaptive feedback, predictive feedback, and neural network feedback, among others. Building upon these control strategies and tailored to different controlled objects, numerous hybrid control algorithms have been derived from the classical PID control algorithm, such as fuzzy PID algorithms and predictive PID algorithms [41,42].

PID feedback control represents a classical strategy in sampled-error feedback control. It applies proportional, integral, and derivative coefficient-weighted multiplications to the error value, ultimately generating a control output that satisfies the desired response. The controller’s output signal regulates the target object until its response aligns with the expected outcome [43,44].

**Figure 1:**
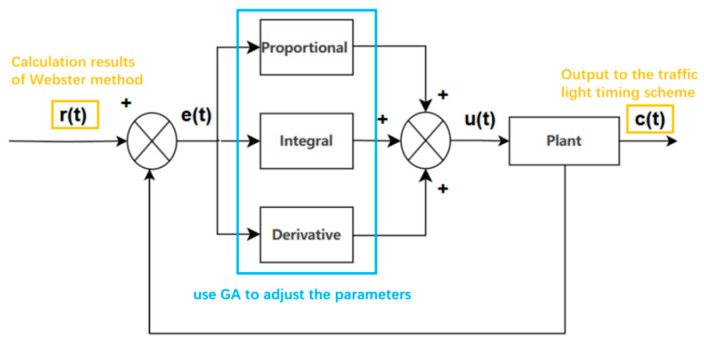
The workflow and structure of how the PID controller adjusts the result of Webster method.

Therefore, we propose a method that combines the Webster timing method with the PID controller. The PID controller, whose parameters are optimized by the genetic algorithm, adjusts the solution obtained by the Webster method to give the final output. The structure of this method is shown in Figure 1.

**Figure 2:**
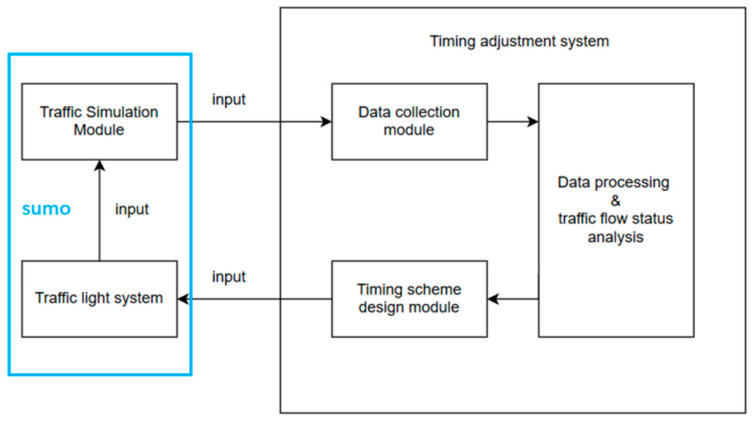
The system’s overall modeling approach and interaction processes.

In Figure 2, r(t) represents the reference input (i.e., the target value or desired output of the system), e(t) denotes the error value (the deviation between the reference input and actual output), u(t) is the control input used to regulate the controlled object, and c(t) is the controlled output—the target variable that needs to be adjusted to approach r(t). The control variable can be expressed by Equation (4).

(4) $$u(t)=k_{p}e(t)+k_{i}\int e(t)dt+k_{d}\frac{\mathrm{de}(t)}{dt},$$

The PID controller mainly consists of three components: the proportional element, integral element, and derivative element.

The proportional element makes the controller’s response proportional to the current error magnitude, rapidly reducing large errors. The integral element eliminates potential steady-state errors by accumulating error values to adjust the control output, ensuring that the system achieves its target value. The derivative element predicts error trends, reducing overshoot and oscillations while improving system stability and response speed.

Through the combination of these three elements and by adjusting their respective parameters, the PID controller can optimize its performance to meet specific application requirements. 

### 2.3. Modeling

**Figure 3:**
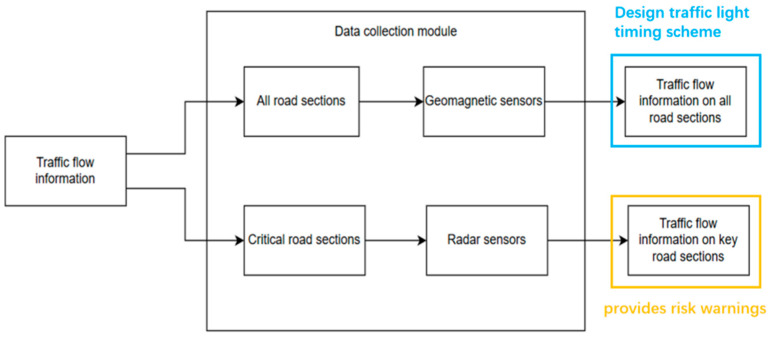
The interaction mechanism between the data collection module and the simulation system, along with the acquired information content.

The traffic signal timing control system comprises three core components—(1) the traffic simulation module, (2) the timing adjustment system, and (3) the traffic signal module—along with their interactive relationships (as illustrated in Figure 3). The timing adjustment system collects traffic data from the simulation module through its embedded data acquisition unit. The raw data undergoes preprocessing and analytical processing in the data processing center before being fed into the timing design module for optimization. The resulting timing schemes are subsequently implemented in the traffic signal system to regulate traffic conditions within the simulation environment.

#### 2.3.1. The System Architecture

A dual-layer sensor network composed of geomagnetic sensors and radar detectors is established to achieve a comprehensive perception of road network traffic status and risk assessment.

The geomagnetic sensors feature a low cost and a small coverage range. Through the uniform deployment and weighted fusion of multi-source data, they support signal timing optimization. The radar sensors exhibit high detection efficiency but have a higher cost and are only deployed in congestion-prone road sections. Based on occupancy rate thresholds (>60% triggers congestion warning, >75% combined with sudden speed reduction triggers accident warning), they enable risk monitoring. These two types of sensors collectively form the network, detecting road information from different perspectives and evaluating the adjusted results.

For traffic signal timing design, different methods are compared to obtain regulation approaches suitable for road sections with various traffic flow characteristics.

The comparison includes fixed timing plans, adaptive regulation, and the Webster method combined with PID controller regulation.

The fixed timing plan acquires traffic flow patterns through manual statistical data and adjusts signal timing accordingly, without subsequent modifications to the timing scheme.

The integration of adaptive control strategies with the Webster method, augmented by a proportional–integral–derivative (PID) controller, offers enhanced flexibility in traffic signal timing optimization. This hybrid approach allows for periodic adjustments based on real-time traffic conditions, ensuring a more dynamic and efficient traffic management system [45,46].

**Figure 4:**
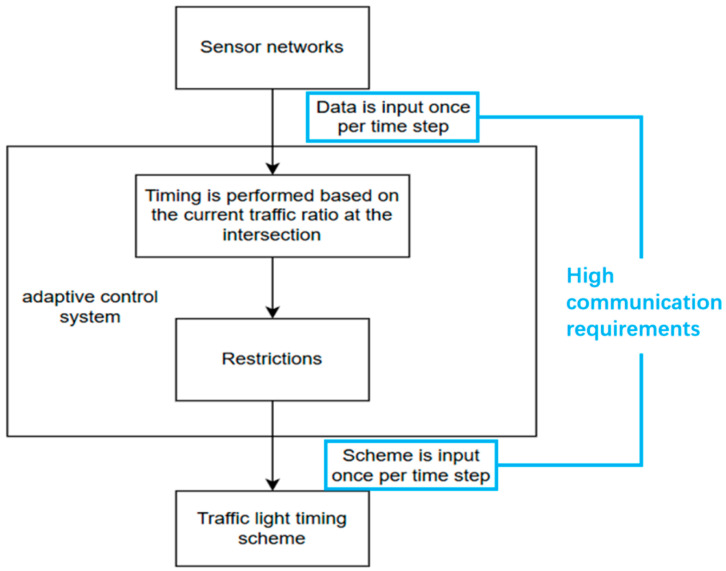
The operational workflow and system architecture of the basic adaptive control system.

The adaptive control framework relies on geomagnetic sensors for continuous traffic flow detection and feedback. By processing real-time sensor data, the system dynamically evaluates the current signal timing plan at each time step and adjusts the green light duration accordingly to optimize traffic throughput. This real-time adaptability enables the system to respond promptly to fluctuations in traffic volume and congestion patterns. However, this mode has high communication requirements when transmitting data. Figure 4 shows the framework of this working mode and its steps with high communication requirements.

In implementing the Webster–PID hybrid control approach, several key assumptions and computational steps are established. First, road sections are initially classified as standard segments, and deceleration effects in turning lanes are disregarded. Second, the lane saturation flow rate is set at 1600 vehicles per hour. Third, time intervals during which no vehicles pass through an intersection are considered as lost time. Fourth, low-traffic-volume periods (≤5 vehicles) are accounted for as 50% lost time. Finally, the initial cycle length and green time allocations are determined using the Webster method (Equations (1) and (2)), while the PID controller subsequently fine-tunes the preliminary cycle to generate the final optimized signal-timing scheme.

For the selection of the PID control strategy, the positional PID algorithm is particularly well-suited for traffic signal optimization [11]. This algorithm iteratively corrects errors between sampled and target values, progressively refining the signal timing plan to better match real-time traffic demands. The input deviation behavior of the PID controller follows the governing Equation (5), ensuring systematic adjustments to improve traffic flow efficiency.

By integrating the predictive capability of the Webster method with the adaptive tuning mechanism of the PID controller, this hybrid approach enhances both the responsiveness and stability of urban traffic signal control systems. It effectively balances traffic flow across intersections, reduces congestion, and improves overall transportation efficiency.

(5) $$u(k)=K_{p}\left(e(t)+\frac{T}{T_{i}}\sum_{n=0}^{k}e(n)+\frac{T_{d}}{T}(e(k)-e(k-1))\right),$$

By transforming Equation (5) into its transfer function representation, we obtain the more interpretable Equation (6).

(6) $$G(s)=\frac{U(s)}{E(s)}=K_{p}(1+\frac{1}{T_{i}s}+T_{d}s),$$

Here, the controller output is denoted as U(s) and the regulator deviation input signal as G(s); $K_{p}$ represents the proportional gain, T is the integral time constant, $T_{d}$ is the derivative time constant, and s is the complex frequency variable in the Laplace transform.

It can be concluded that the adjustment magnitude during each control cycle is determined by three parameters: proportional, integral, and derivative terms. Through systematic optimization methods, the most suitable control coefficients for the current system can be identified, thereby achieving optimal controller performance.

After preliminary calculations and subsequent fine-tuning, the final traffic signal timing cycle can be determined.

#### 2.3.2. k-Means Dynamic Weights, GA-Optimized PID Controller Parameters

Data is collected through sensors. Each intersection has two indicators used to quantify whether the intersection is standard, namely, the intersection speed and road occupancy rate. In the case of N (N > 2) intersections, the speed and road occupancy rate of each intersection are Z-score standardized to obtain standardized data. The speed and occupancy rate used for calculation are the average values of the values in the four directions of the intersection:

(7) $${\upsilon^{\prime }}_{j}=\frac{\upsilon_{j}-\mu_{\upsilon }}{\sigma_{\upsilon }},$$

(8) $${\;o\;\prime }_{j}=\frac{o_{j}-\mu_{o}}{\sigma_{o}},$$

$\mu_{\upsilon }$ and $\sigma_{\upsilon }$ are the mean and standard deviation of vehicle speed, respectively, and $\mu_{o}$ and $\sigma_{o}$ are the mean and standard deviation of road occupancy, respectively.

First, determine the intersection with the lowest speed and the highest road occupancy, and then use the k-means method to determine the 20% (rounded) intersections, with traffic lights close to this intersection as “more important” intersections. In more complex intersections, more clusters need to be divided for planning.

For a single cluster, first calculate the index. The larger the D value, the worse the traffic situation.

(9) $$D_{k}=\sum_{j\in C_{k}}\frac{1}{1+{\upsilon^{\prime }}_{j}}\times {o^{\prime }}_{j},$$

where $C_{k}$ represents the *k*th cluster, which is dynamically calculated based on the ratio of the flow density of each cluster to the sum of the flow density of all clusters and can reflect the impact of the current intersection on the overall traffic flow:

(10) $$\omega_{k}=\frac{D_{k}}{\sum_{i=1}^{k}D_{i}},$$

$D_{i}$ represents the traffic condition of intersection i, and $\omega_{i}$ represents the weight value of intersection i.

The sensors can be used to obtain the occupancy rate of each road at multiple time points, the queue length U of 12 sections in two directions, and the total queue length of each intersection:

(11) $$U=-\sum_{j=1}^{4}L_{j},$$

The total queue length of the road network can be obtained by summing the total lengths of the two intersections, where $L_{j}$ is the queue length of the intersection in a certain direction.

Because traffic flow and occupancy rate change dynamically, the weight coefficient needs to be adjusted regularly to cope with the traffic flow changes at the intersection, and the adjustment is made every 500 time steps (the adjustment interval is designed according to the conditions of the specific road section).

Accordingly, after determining the different weights of each intersection, combined with the traffic flow of each intersection and the determined adaptability function, it can be written as follows:

(12) $$A_{1}=\frac{1}{1+\sum_{i\in C_{impor\tan t}}^{i}\omega_{i}U_{i}},$$

(13) $$A_{2}=\frac{1}{1+\sum_{i\in C_{impor\tan t}}^{i}\omega_{i}U_{i}+\sum_{j\in C_{normal}}^{j}\omega_{j}U_{j}},$$

$A_{1}$ represents the fitness function of the main intersection, and $A_{2}$ represents the fitness function of all intersections. When adjusting with the genetic algorithm, the fitness of the main intersection is ensured first.

When using the genetic algorithm for tuning, first, the PID controller of each intersection is regarded as an independent individual, and the genetic algorithm is used to optimize the parameters separately, and the traffic flow in the period of 2500–4000 is selected as a sample.

In order to increase the coordination between each intersection, all PID controllers in the road network are regarded as a whole, and the genetic algorithm is used for unified optimization to enhance the coordinated control between intersections. The PID parameter combination of the intersection is used as a chromosome, and the traffic flow in the period of 2500–4000 is selected for training. When adjusting the parameters, 20% of the random points are selected for crossover, and 10% of the random points are selected for random changes within the range of 10% of the original parameters, and the optimal result is taken for iteration. In order to ensure that the optimization process does not affect the control of traffic signals, the number of iterations is controlled at 500 time steps. Finally, a new PID parameter group is derived based on the overall road network and input.

When dealing with more complex road networks, the k-means method can be used to determine the initial weight value, and then PSO can be used to optimize it. The initial value given by the k-means method can ensure that the system can at least obtain the local optimal solution and that the PSO can get closer to the global optimal solution, making the system have better convergence and stability.

#### 2.3.3. Road Environment Modeling

**Figure 5:**
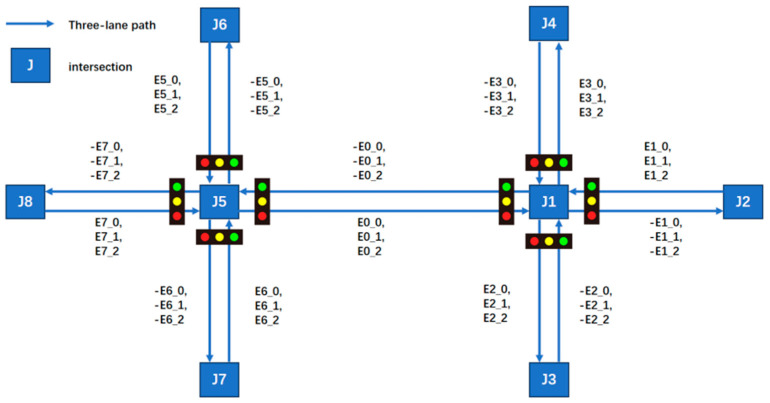
The road infrastructure configuration.

The experimental road network model, as illustrated in the Figure 5, consists of two interconnected signalized intersections. The network is composed of eight nodes, each configured with three-lane approaches, including designated through lanes, left-turn lanes, and right-turn lanes, without additional features such as roundabouts. The segment connecting J1 and J5 spans 200 unit lengths, while all other segments measure 100 unit lengths.

To accurately replicate peak-hour traffic variations, the vehicle generation process follows a high–low–high distribution pattern. This pattern ensures that traffic demand dynamically fluctuates, mimicking real-world peak congestion scenarios.

In the initial phase (time steps 0–3600), 70% of all approaches are randomly selected, each generating vehicles at an average rate of 100 vehicles per hour. This phase represents relatively moderate traffic flow before peak congestion begins. During the peak period (time steps 3600–5400), the base traffic volume remains at 100 vehicles per hour for 70% of approaches. However, to simulate high congestion levels, critical approaches receive an additional 350 vehicles per hour, ensuring that traffic flow reaches 85–90% of road capacity without causing complete gridlock. In the post-peak phase (time steps 5400–9600), the proportion of active approaches increases to 80%, with an average generation rate of 150 vehicles per hour per approach. This phase reflects the gradual dissipation of peak congestion, transitioning toward normal traffic conditions.

This structured traffic demand model effectively captures dynamic fluctuations observed in real-world peak-hour conditions. It provides a realistic framework for evaluating traffic control strategies and assessing system performance under varying congestion levels.

**Figure 6:**
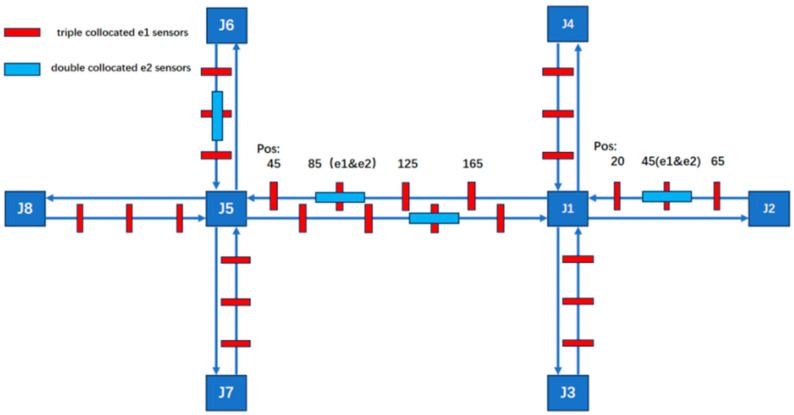
The sensor network composed of heterogeneous sensors within the road network.

All possible sensor types and their locations are shown in Figure 6. Geomagnetic sensors are used to detect all road sections, and radar sensors are used to detect key road sections. The critical segments include J5–J6, through and left-turn movements; J1–J5 through movements; and J1–J2, through and right-turn movements.

## 3. Results

### 3.1. Sensor Network

To ensure comprehensive detection under consistent traffic flow conditions, both the quantity and positioning of sensors were systematically optimized. The experimental setup took into account real-world driving behaviors, such as lane-changing maneuvers and the presence of dedicated turning lanes, to more accurately replicate actual traffic scenarios. To guarantee uniform detection across all lanes and ensure the robustness of the testing methodology, sensors were strategically deployed in each individual lane, thereby facilitating the collection of complete and accurate traffic data for every lane of the road network. Additionally, to assess the sensor network’s performance under high-volume traffic conditions, the optimization process was validated using raw, unadjusted data collected from fixed-time signal control schemes. This approach provided an empirical basis for evaluating the effectiveness and reliability of the sensor network during periods of peak traffic flow, ensuring that the system could handle the complexities of real-world traffic congestion.

#### 3.1.1. Sensor Deployment Configuration for Scenario 1:40–1:33

**Figure 7:**
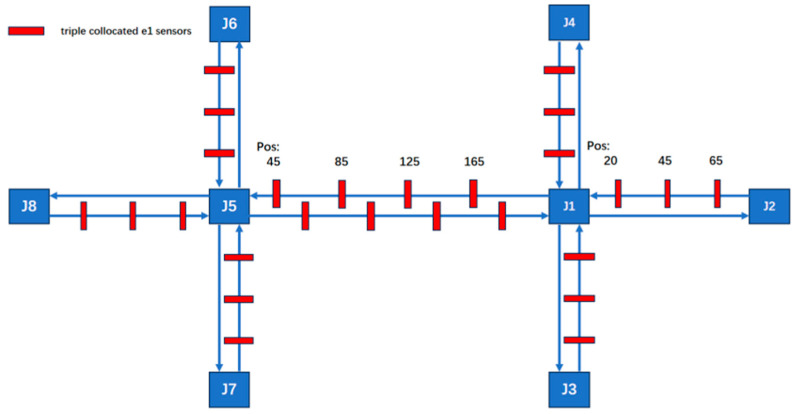
The arrangement of 78 sensors.

By adopting the layout of all 78 sensors, 3 sensors are placed on a 100-unit road and 4 sensors on a 200-unit road. At this time, the coverage of the entire road section by the deployed sensors accounts for 10% to 20% of the entire road section, and the ratio of sensors to road length is 1:40 to 1:33. The deployment of sensors is shown in Figure 7.

The deployment positions are at the exit of the road section, the middle of the road section, and 80% of the road section. This can ensure detection even in cases of non-severe congestion that does not paralyze the road network.

**Figure 8:**
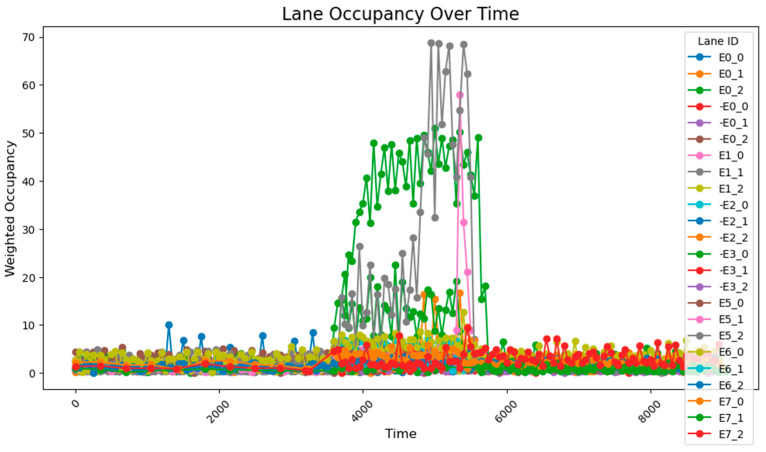
The data acquisition results from the sensor network consisting of 78 units.

The data collected following the simulation, as shown in Figure 8, clearly illustrate the variation in traffic volumes across different time periods. During time steps 0–3800, traffic volumes were relatively low, with overall occupancy rates remaining between 0 and 10%. From time steps 3800–5800, traffic volumes increased significantly, exhibiting two distinct trends: gradual accumulation and sustained high values. A comparison between these segments and those receiving higher input flows reveals that segments with cumulative increases received traffic from both current high-flow sources and previous low-flow sources. These segments experienced slower accumulation due to the longer distance between the entry points and the accumulating segments. By contrast, segments maintaining stable high values primarily received traffic from high-flow sources during peak periods and were located closer to the flow entry points. During time steps 5800–9600, traffic volumes once again decreased, returning to lower levels. The road occupancy rate collected by the sensor is shown in Figure 8.

Considering that occupancy rates typically lag behind actual flow inputs, coupled with the precise input volumes and heavily loaded segments, the sensor deployment configuration proves to be both reasonable and effective for comprehensive data collection. The flow range and distribution patterns of the collected data are fundamentally consistent with the predicted flow characteristics based on the input data, with minor deviations primarily attributed to short sampling durations. These fluctuations remain within a stable range and do not interfere with the data processing or analysis. Therefore, it can be concluded that the data collected after subsequent adjustments to the sensor network should be compared with this dataset, with the aim of ensuring data completeness while minimizing the number of sensors required.

#### 3.1.2. Sensor Deployment Configuration for Scenario 1:200–1:100

**Figure 9:**
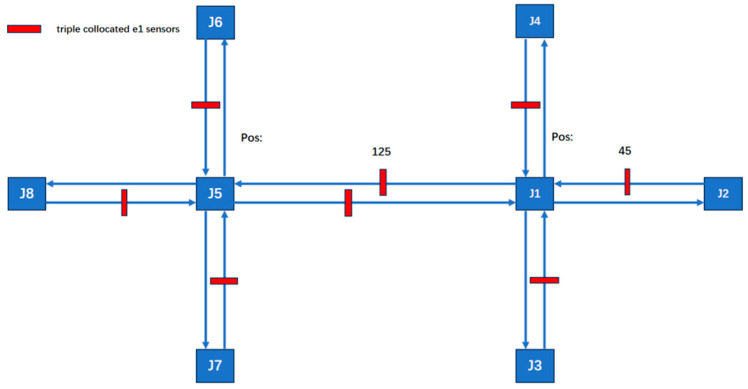
The arrangement of 24 sensors.

The sensor deployment adopts a configuration with one sensor per road segment, maintaining a sensor-to-segment-length ratio of 1:100 for short segments and 1:200 for long segments. Compared to the 78-sensor network, this arrangement achieves a 69% reduction in sensor quantity. The deployment of sensors is shown in Figure 9.

After running the simulation, the collected data reveal that during time steps 0–3800, the results are largely consistent with those obtained using the 78-sensor network. However, during time steps 3800–5800, when traffic flow was relatively high, two distinct trends emerged: gradual accumulation and sustained high values.

**Figure 10:**
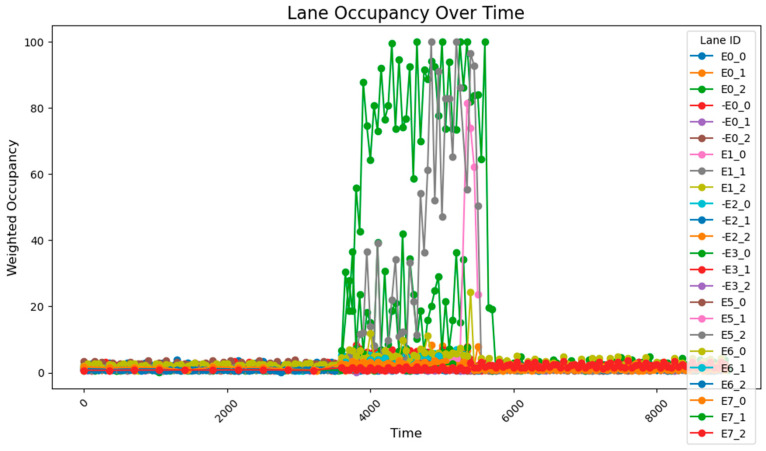
The data acquisition results from the sensor network consisting of 24 units.

A comparison with standard reference data shows that the 24-sensor network fails to accurately capture values under high-volume traffic conditions. In the single-sensor configuration, detection is confined to a 5-unit coverage area around each sensor, which limits the system’s ability to track vehicle movements in sensor-blind zones. This results in erroneous calculations, particularly when traffic congestion occurs mid-segment; the system may incorrectly report 100% blockage across the entire segment. The road occupancy rate collected by the sensor is shown in Figure 10.

These findings underscore the impracticality of deploying sensors at a ratio of 1:100 or lower, as this configuration fails to capture accurate data during periods of increased traffic flow and leads to systemic calculation errors. Consequently, the sensor deployment strategy must be revised by increasing the total number of sensors and optimizing their spatial distribution to ensure comprehensive and accurate monitoring of traffic flow across all roadway segments.

#### 3.1.3. Sensor Deployment Configuration for Scenario 1:66–1:50

**Figure 11:**
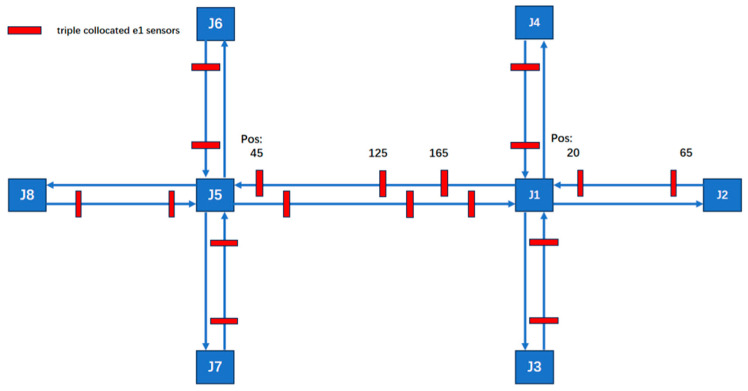
The arrangement of 54 sensors.

Considering the correlation between road length and sensor quantity, the deployment strategy has been adjusted to incorporate three sensors per long road segment. This results in sensor-to-road-length ratios of 1:50 for long segments and 1:66.6 for short segments. Compared to the original 78-sensor network, this optimized configuration requires only 54 sensors, achieving a 30% reduction in total sensor count while maintaining effective coverage. The deployment of sensors is shown in Figure 11.

**Figure 12:**
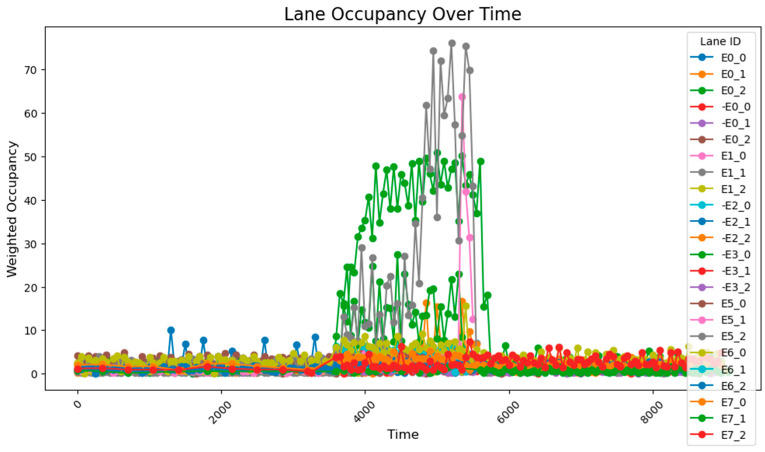
The data acquisition results from the sensor network consisting of 54 units.

After conducting the simulation, the collected data reveal that during time steps 0–3800, the results align closely with those obtained using the 78-sensor network. During time steps 3800–5800, when traffic flow was relatively high, two distinct trends were observed: gradual accumulation and sustained high values. The road occupancy rate collected by the sensor is shown in Figure 12.

A comparison with standard reference data indicates that the 54-sensor network effectively maintains accurate detection capability, even during sudden traffic surges. Moreover, the data collected during time steps 5800–9600 exhibit fundamental consistency with the expected conditions, further validating the reliability of the 54-sensor configuration.

#### 3.1.4. Sensor Deployment Configuration for Scenario 1:100

**Figure 13:**
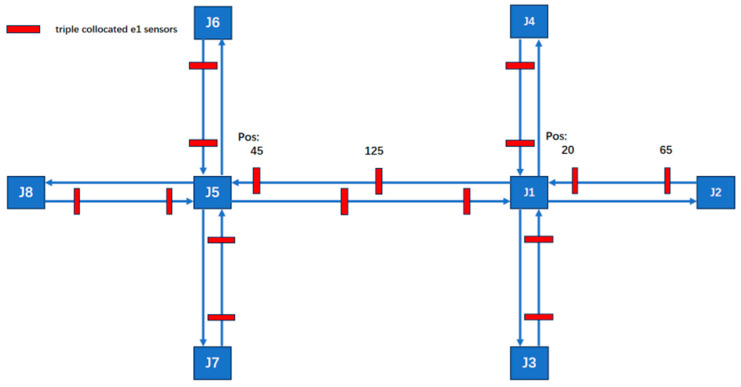
The arrangement of 48 sensors.

Specifically, to validate that an approximate 1:50 deployment ratio ensures detection accuracy (as opposed to simply deploying two or more sensors per roadway), a new road network configuration was implemented. The layout deployed 2 sensors per 100-unit road segment and 2 sensors per 200-unit road segment, resulting in a 48-sensor network. To specify by segment length, 2 sensors were deployed per 100-unit segment and 2 sensors per 200-unit segment, constituting a network configuration of 48 sensors total. The deployment of sensors is shown in Figure 13.

**Figure 14:**
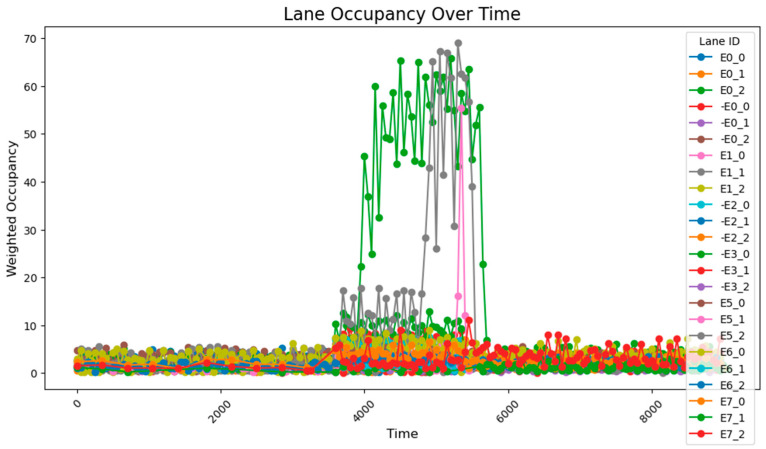
The data acquisition results for the sensor network consisting of 48 units.

After running the simulation, the collected data show that during the 0–3800 time period, the results are fundamentally consistent with those obtained using the 78-sensor network. However, during the 3800–5800 time period, certain segment flow rates were still miscalculated. This repeated occurrence of flow calculation errors during the 3800–5800 period demonstrates that two sensors are insufficient to capture all data characteristics, proving that a 1:100 deployment ratio is inadequate, while a sensor deployment rate of approximately 1:50 is relatively more effective. The road occupancy rate collected by the sensor is shown in Figure 14.

### 3.2. Timing Plan Selection and Optimization

#### 3.2.1. Explanation of the Measurement Criteria

**Figure 15:**
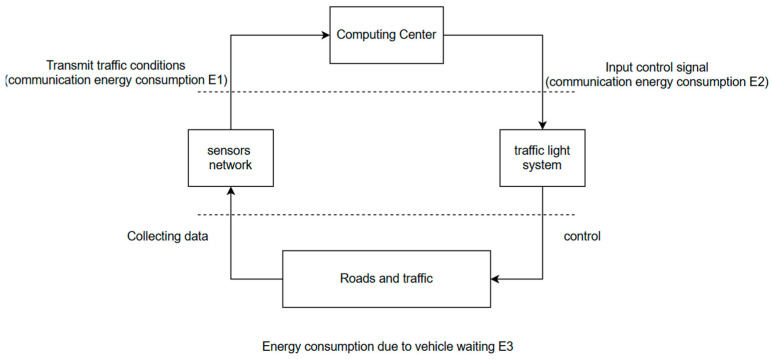
The energy consumption of the whole system.

In the entire traffic system, communication energy consumption E1 is generated when sensors collect data and send it to the computing center, and communication energy consumption E2 is generated when the computing center sends control signals to the traffic light system. The energy consumption generated by cars waiting on the road is called E3. While the signal is being optimized, E2 is reduced by improving the interval of sending information. The structure of the system and an illustration of how the system generates energy consumption are shown in Figure 15.

In the image showing the results, the y-axis is represented by occupancy rate, which means the ratio of the time that a vehicle occupies the sensor to the detection time during a period of detection. The queue length $L_{\mathrm{queue}}$ can be calculated by the occupancy rate ρ and the lane length $L_{\mathrm{lane}}$. However, considering that not all roads in the road network are of the same length, in order to ensure the uniformity of the measurement standard, the occupancy rate is used to represent the traffic level of the road network. The lower the occupancy rate, the higher the traffic level.

(14) $$L_{\mathrm{queue}}=\rho \times L_{\mathrm{lane}}$$

#### 3.2.2. Unregulated

According to SUMO’s default settings, the timing for the four phases of traffic lights J1 and J5 is as shown in Table 3, with J1 and J5 maintaining identical configurations.

**Figure 16:**
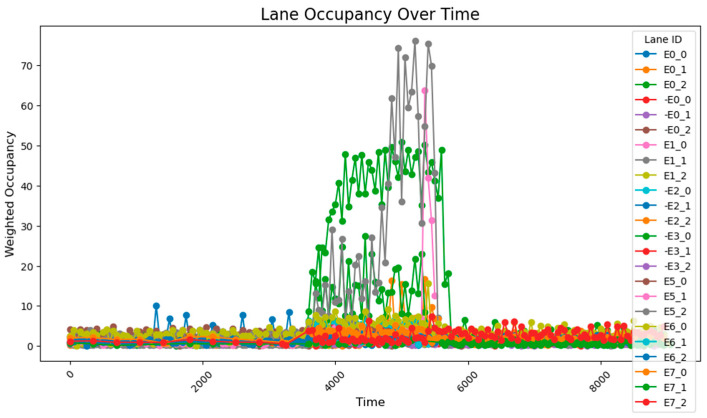
The unadjusted road occupancy conditions.

Under unadjusted conditions, the data collected using the designed sensor network reveal the following patterns: during time steps 3800–5800, road E0_2 exhibited a gradual increase and stabilized at approximately 50% occupancy; and roads E1_1 and E1_0 progressively accumulated to peak values of about 70% and 60%, respectively. The road occupancy rate collected by the sensor is shown in Figure 16.

Statistical calculations of data across all time periods show an average road congestion rate of 1.97% overall, with key segments demonstrating an average occupancy rate of 23.3% and an average congestion rate of 5.20% during peak hours.

#### 3.2.3. Fixed-Time Control

First, the input traffic flow during peak hours is statistically analyzed for each road segment. The green time allocation for the two intersections J1 and J5 is then determined while keeping the total cycle length constant. Although this method does not account for potential cumulative traffic effects, it effectively alleviates the congestion pressure on busy roads compared to simple equal distribution. The adjusted phase timing for traffic lights at J1 and J5 is shown in Table 4 and Table 5, with the total green time set to 80 units. The allocation ratios are adjusted based on the statistically derived traffic flow data.

After a simple adjustment based on the proportion of traffic at each intersection during peak hours, it was observed that road congestion significantly improved. During the time steps 3800–5800, the occupancy rate on the E0_2 road remained around 30%, peaking at approximately 37%, while the occupancy of the E1_1 and E1_0 roads remained relatively stable at 15%. However, this improvement came at the cost of increasing the occupancy rate of the E0_0 road to about 12%. The average occupancy across all road sections was 1.50%, while during the peak period (time steps 3800–5800), the occupancy of key road sections rose to 7.51%, with the overall occupancy across all sections reaching 2.97%.

**Figure 17:**
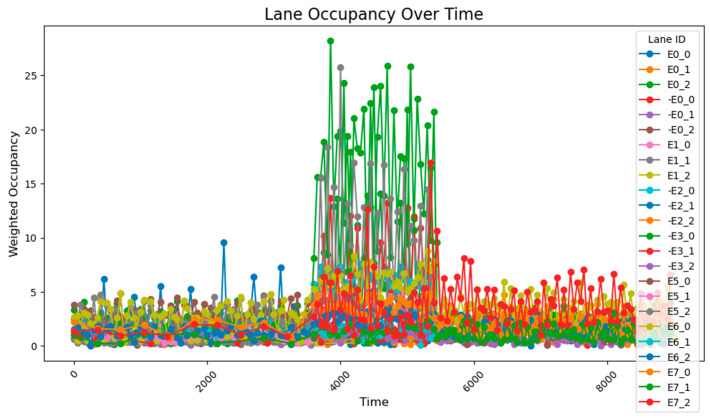
The road occupancy after proportional adjustment.

Compared to the unadjusted scenario, the simple timing adjustments proved effective in managing relatively stable traffic fluctuations (especially between time steps 3800 and 5800). However, this method may lead to excessive waiting times for some road sections. Simultaneously, the occupancy rate of the higher traffic roads was maintained between 15% and 25%. The road occupancy rate collected by the sensor is shown in Figure 17.

#### 3.2.4. Fully Adaptive Control Based on Sensor Data

**Figure 18:**
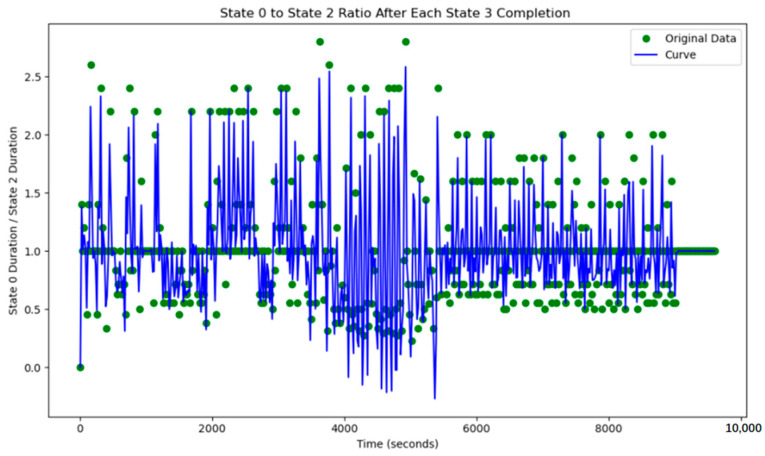
The north–south to east–west green time ratio at J1 under adaptive control.

**Figure 19:**
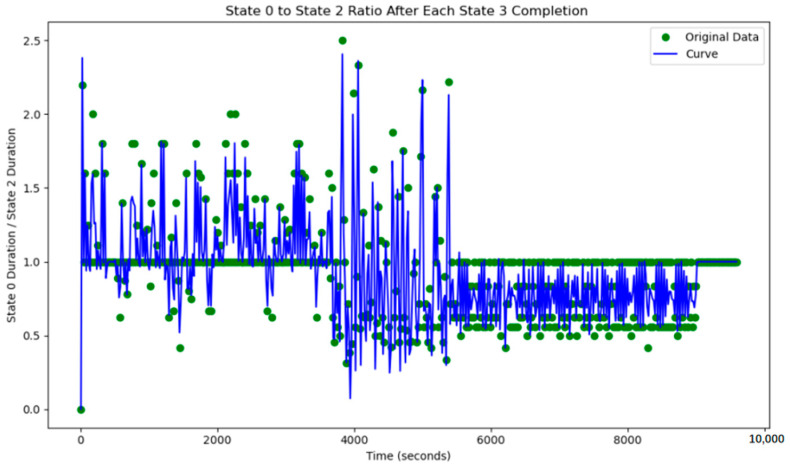
The north–south to east–west green time ratio at J5 under adaptive control.

This simple adaptive control strategy dynamically adjusts the corresponding green time based on real-time traffic flow variations detected at each intersection. At every time step, the system performs verification and modification of the current timing plan. The variation of the green light cycle is shown in Figure 18 and Figure 19.

After implementing a simple adaptive adjustment method, a significant improvement in congestion during peak hours was observed, with a notable enhancement in the balance of occupancy rates across various intersections. Through coordinated adjustments, the occupancy distribution across all road segments during time steps 3800–5800 became more concentrated, with the average occupancy rates of each segment ranging from 2.5% to 12.5%.

The overall occupancy rate across all road segments, measured over all time steps, was 1.30%. During the peak hours (time steps 3800–5800), the occupancy rate of key road segments was 5.80%, while the average occupancy rate for all segments was 2.56%. The simple adaptive adjustment method effectively alleviated road congestion and, according to reports from the risk monitoring network, also played a crucial role in preventing both congestion and associated road risks.

**Figure 20:**
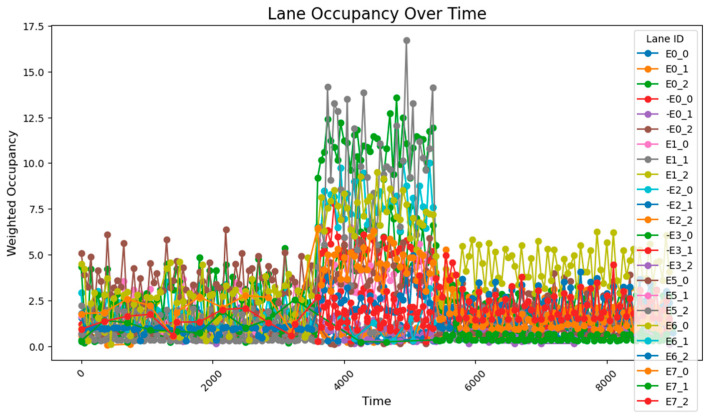
Illustrates the road occupancy after a simple adaptive adjustment.

However, based on the lane flow data from time steps 3800 to 5800, it is evident that many previously low-traffic roads have experienced a substantial increase in occupancy rates. This suggests that the adaptive adjustment method may disproportionately utilize roads with lower traffic volumes. Additionally, adjustments made to intersections J1 and J5 reveal that performing updates at every single time step is too frequent, resulting in high energy consumption. The method also requires continuous data transmission over short intervals, which places significant demands on data transfer capacity and communication stability. Such frequent transmission increases the likelihood of errors due to potential interference. Moreover, looking at the major adjustment points in the graph, the shortest interval between significant adjustments is found to range from 500 to 1000 time steps. This provides a useful reference for optimizing the adjustment cycle in subsequent methods. The road occupancy rate collected by the sensor is shown in Figure 20.

#### 3.2.5. Optimization of the Webster Method and PID Controller and Comparison with Advanced Methods

This method employs two regulation rhythms, adjusting every 1000 and 2000 time steps, to determine which mode performs better. From the occupancy change graphs adjusted using these two methods, it is evident that while there is still a peak during peak hours, the risk-warning sensor network is not triggered. In E0_2 and E0_0, multiple peaks of 30–50% are observed, while E1_1 remains generally stable at around 10%. The occupancy rate of less-trafficked road sections is maintained below 10%, with limited exploitation of these sections.

Additionally, the frequency of adjustments was plotted, demonstrating that adaptive control could achieve nearly the same, or even better, results with lower energy consumption.

**Figure 21:**
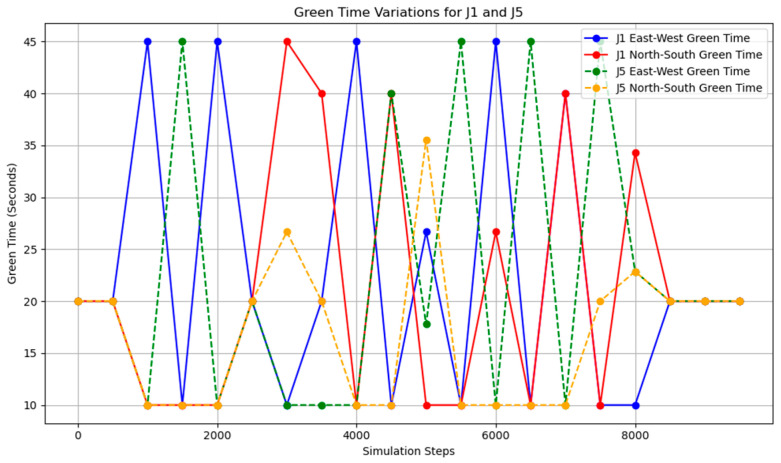
The change in the duration of the green light after adjusting intersections J1 and J5 every 500 time steps.

**Figure 22:**
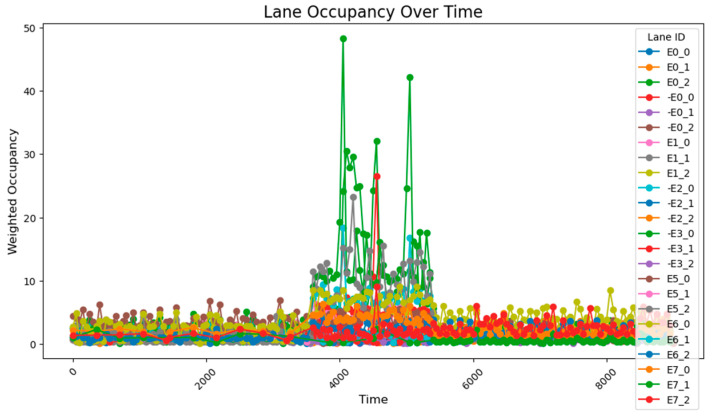
The road occupancy rate is adjusted every 500 time steps by Webster method and PID controller.

When adjusting every 500 time steps, the average occupancy across all road segments was 1.29%. For the time step range of 3800–5800 (peak hours), the occupancy rate of key road sections was 6.35%, while the occupancy rate of all road sections was 2.66%. When adjusting every 1000 time steps, the average occupancy across all segments was 1.27%, the occupancy rate of key road sections during the peak hours was 7.73%, and the overall occupancy rate was 2.77%. The change of green light cycle is shown in Figure 21. The road occupancy rate collected by the sensor is shown in Figure 22.

**Figure 23:**
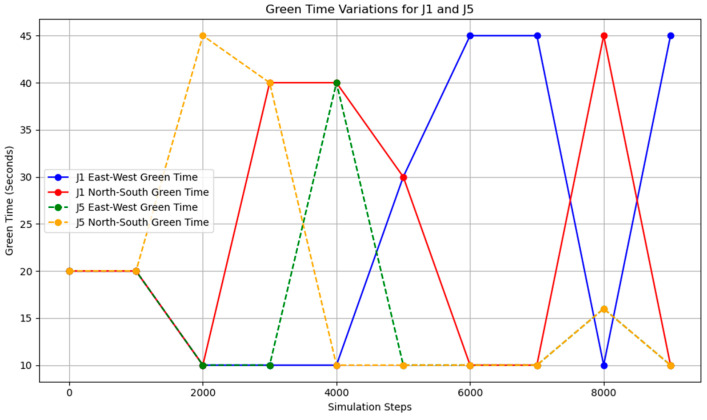
The change in the duration of the green light after adjusting intersections J1 and J5 every 1000 time steps.

**Figure 24:**
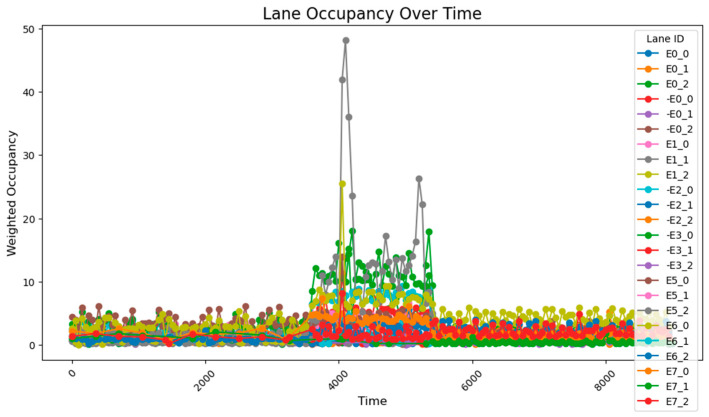
The road occupancy rate is adjusted every 1000 time steps by Webster method and PID controller.

Compared to the adjustment made every 500 time steps, the occupancy rate of all road sections decreased by 0.02% when adjusting every 1000 time steps, but during peak hours, the occupancy of key road sections and all road sections increased by 1.38% and 0.11%, respectively. These findings suggest that while the 1000-time-step interval provides a better overall adjustment effect for the road network, the effectiveness of the adjustment during peak times and for key roads is somewhat reduced. The change of green light cycle is shown in Figure 23. The road occupancy rate collected by the sensor is shown in Figure 24.

**Figure 25:**
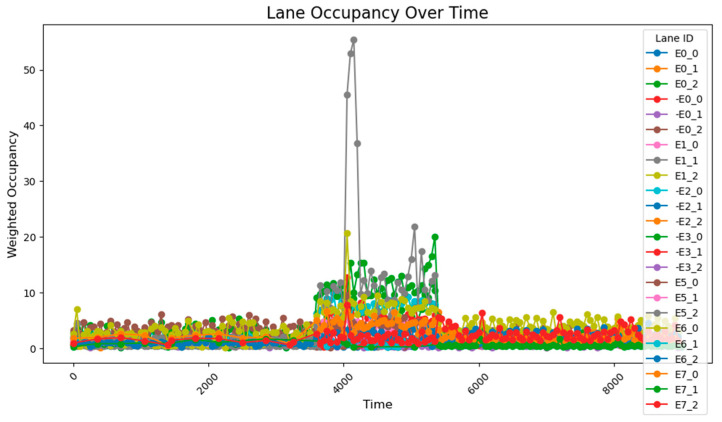
The road occupancy rate is adjusted every 1000 time steps by Webster method and PID controller with trained parameters.

When utilizing the trained parameters, the average occupancy rate across all road sections at all times is 1.24%. During the critical time steps (3800–5800), the occupancy rate for key road sections is 6.56%, while the rate for all sections is 2.60%. Compared to the controller with untrained parameters (adjustments made every 1000 time steps), the overall occupancy rate decreased by 0.02%, the occupancy rate for key sections during critical times dropped by 1.07%, and the occupancy rate for all sections during critical times fell by 0.17%. Additionally, the performance across all road sections during the critical time steps was superior to that observed with untrained parameters and adjustments made every 500 time steps. The road occupancy rate collected by the sensor is shown in Figure 25.

Furthermore, the risk monitoring network did not trigger any warnings for congestion or accident risks during the period.

**Figure 26:**
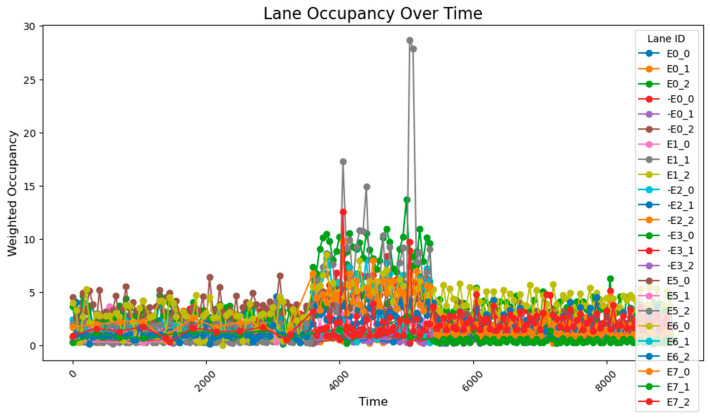
The road occupancy rate adjusted every 1000 time steps by the Webster method with the overall PID parameter control optimized by the GA algorithm.

These findings suggest that the PID controller with optimized parameters has significantly improved performance compared to its original configuration, offering more efficient traffic management and enhanced risk mitigation. The road occupancy rate collected by the sensor is shown in Figure 26.

**Figure 27:**
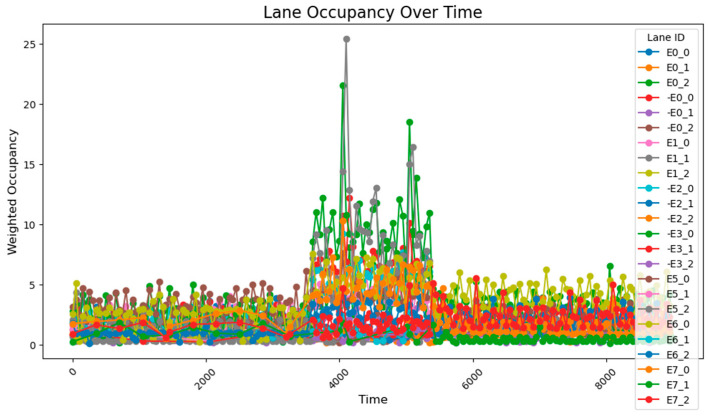
The road occupancy rate adjusted every 1000 time steps by the PSO method.

After using the PID parameter group adjusted according to the overall situation of the road network, the average occupancy rate of all sections at all times is 1.20%; the occupancy rate of key sections between 3800 and 5800 time steps is 4.49%, and the occupancy rate of all sections is 2.15%, which is lower than the previous best result. It can be seen that after using the genetic algorithm to perform global optimization of the PID parameters, the efficiency of road traffic has been further improved. The road occupancy rate collected by the sensor is shown in Figure 27.

In order to increase the comparison with advanced optimization methods, the comparison using the particle swarm algorithm was added. After optimization using the particle swarm method, the average occupancy rate of all sections at all times was 1.25%; the occupancy rate of key sections between 3800 and 5800 time steps was 4.97%, and the occupancy rate of all sections was 2.27%. The effect of the particle swarm algorithm is better than that of almost all algorithms, and the effect is almost the same as the method of using GA to optimize the PID controller at each intersection separately, but it is better than it in controlling the peak value.

#### 3.2.6. Verification in Complex Road Networks

**Figure 28:**
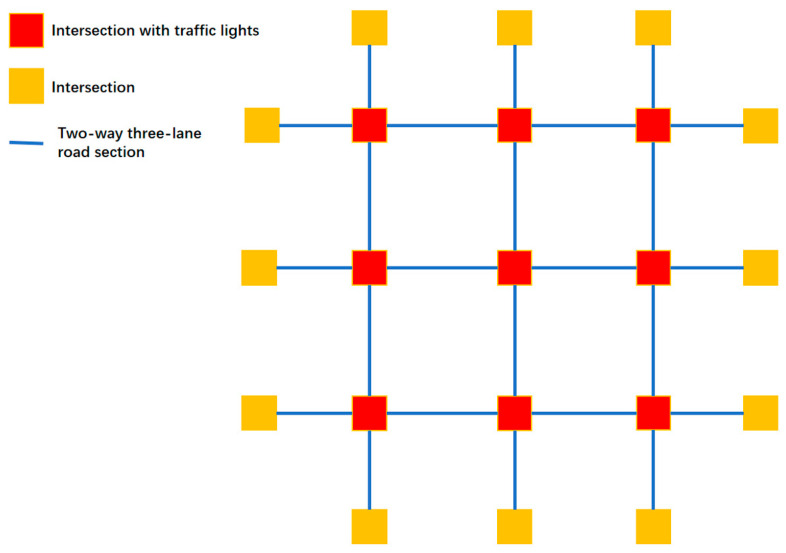
The structure of a more complex road network.

The road network structure in the experiment is a 3 × 3 structure, which includes nine intersections, each with a traffic light. The more complex road network structure is shown in Figure 28.

First, it is stipulated that the change in traffic flow is a low–high–low trend change, and the traffic flow is randomly released in each time period.

**Figure 29:**
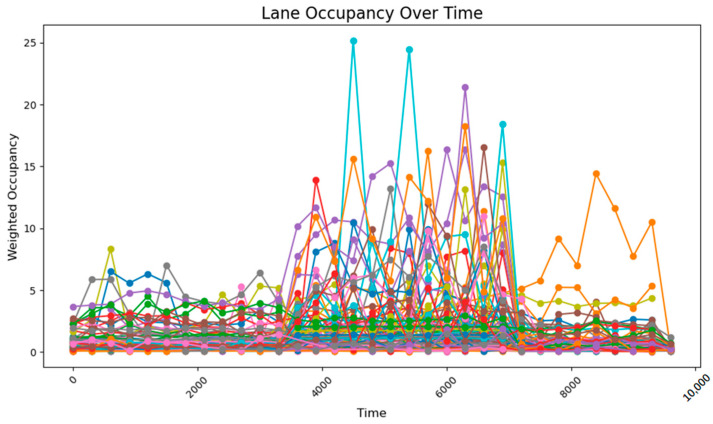
The road occupancy rate without dynamic weight adjustment.

When the key intersections are not changed, the occupancy rate of all sections in the section changes as follows, the road occupancy rate collected by the sensor is shown in Figure 29.

**Figure 30:**
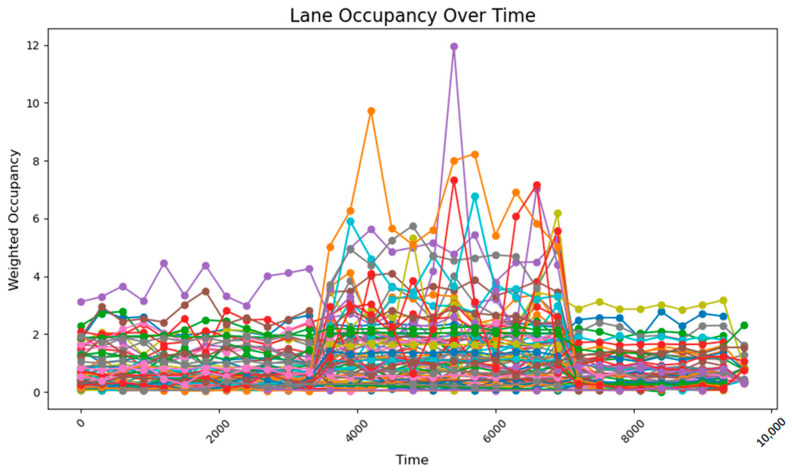
The road occupancy rate with dynamic weight adjustment.

The peak lane occupancy rate in all time periods is 25%, and after the peak period, some sections will experience an unreasonable increase in lane occupancy rate due to previous suboptimal designs. The road occupancy rate collected by the sensor is shown in Figure 30.

After adopting the method of real-time updating of key intersections and adjusting weight values, in a more complex road network, the overall road occupancy rate and peak value are first reduced. At the same time, after the peak period, the occupancy rate does not increase due to the recalculation of weights. This shows that the method of calculating road weight values in real time using k-means can better adjust the timing plan of traffic lights, reduce the road occupancy rate, and improve traffic efficiency when the road network is complex and the traffic volume is random.

## 4. Discussion

Through a comprehensive comparison of different sensor network layouts, we determined that the length of road segments should align with the number of sensors deployed to optimize both energy consumption and data accuracy. Specifically, a sensor deployment ratio of 1:50 to 1:60 effectively reduces energy consumption while maintaining the integrity and accuracy of traffic data. Having successfully captured the traffic flow characteristics, we explored various traffic light timing-adjustment methods, each exhibiting distinct advantages and limitations.

While the fixed-timing scheme can mitigate traffic congestion to a certain extent, it fails to balance the flow across lanes and respond effectively to traffic fluctuations. The adaptive control strategy, based entirely on sensor data, offers similar outcomes to the combined Webster method and PID controller but demands a very high communication frequency and incurs substantial costs for rapid detection and real-time adjustments. Under these two timing schemes, the road occupancy rate during peak hours was reduced by about 1.7%, but the method that combined the Webster method and PID controller has less energy consumption. By contrast, the combination of the Webster method and PID controller operates at a lower adjustment frequency and, despite minor fluctuations during the initial adjustment phase, demonstrates superior performance in maintaining overall road occupancy rates and controlling peak occupancy during most periods. Additionally, fine-tuning the PID controller parameters using a genetic algorithm significantly enhances the overall performance of the model. Among them, the enhancement of the unified optimization method of all PID controllers through the genetic algorithm is more obvious, and the performance exceeds that of the particle swarm algorithm, and the road occupancy rate is further reduced by 0.48% during the peak. Dynamically allocating intersection weights using the k-means method can help to better adapt to situations where the road network is more complex and the traffic flow is more random.

## 5. Conclusions

Optimal Sensor Network Deployment Ratios: By evaluating various sensor network deployment strategies while balancing information perception accuracy and energy consumption for data transmission, we determined that a deployment ratio ranging from 1:50 to 1:60 effectively ensures precise road condition monitoring. This ratio strikes a balance between coverage density and operational efficiency, enabling reliable traffic data collection without incurring excessive energy costs.

Traffic Control Strategies Under Varying Conditions:

Under stable traffic conditions, fixed-timing traffic signal schemes demonstrate the highest energy efficiency, as they minimize unnecessary adjustments and maintain consistent operational cycles.

Conversely, in environments marked by frequent traffic flow fluctuations, adaptive control methods exhibit superior responsiveness. Specifically, the integration of the Webster method with a parameter-optimized PID controller enhances the adaptability of traffic light timing schemes. This approach enables precise adjustments in response to sudden, significant increases in traffic volume, thereby improving overall traffic management effectiveness.

Looking ahead, we plan to explore more complex traffic patterns and mixed traffic flow dynamics (for example, integrating pedestrian, cyclist, and vehicle data), as well as explore and further improve this innovative approach on more complex road networks. Our goal is to improve the performance of the model and ensure that it can provide more accurate and efficient solutions to urban traffic management challenges.

## Figures and Tables

**Table 1:** The costs associated with common sensors (unit: Chinese Yuan per year, ¥/YR).

Sensor Classification	Capital Expenditure	Operational Expenditure	Operational Lifetime	Total Cost of Ownership
Inductive loop detector	750	600	10	1810
Geomagnetic sensor	450	200	10	890
Continuous wave radar	995	3200	7	2130
Frequency-modulated continuous wave radar	3300	400	7	1370
Active infrared system	6000	3200	7	7130
Passive infrared system	700	1200	7	1500
Video image processing system	735	400	10	1730
Ultrasonic sensor	3500	800	7	510

**Table 2:** The performance associated with common sensors.

Sensor Classification	Deployment Location	Counting Error Rate (%)	Speed Measurement Error Rate (%)
Inductive loop detector	Paved surface	3	1.2–3.3
Geomagnetic sensor	Paved surface	2.5	1.4–4.8
Continuous wave radar	Over roadway	2.5–13.8	1
Frequency-modulated continuous wave radar	Over roadway	2	7.9
Active infrared system	Over roadway	0.7	5.8
Passive infrared system	Over roadway	10	10.8
Video image processing system	Over roadway/Roadside	5	2.5–8

**Table 3:** The unadjusted phase-timing configurations for intersections J1 and J5.

Phase	Time (Step)
GGGGgrrrrrGGGGgrrrrr	42
yyyyyrrrrryyyyyrrrrr	3
rrrrrGGGGgrrrrrGGGGg	42
rrrrryyyyyrrrrryyyyy	3

**Table 4:** Adjusted timing scheme for intersection J1.

Phase	Time (Step)
GGGGgrrrrrGGGGgrrrrr	50
yyyyyrrrrryyyyyrrrrr	3
rrrrrGGGGgrrrrrGGGGg	30
rrrrryyyyyrrrrryyyyy	3

**Table 5:** Adjusted timing scheme for intersection J5.

Phase	Time (Step)
GGGGgrrrrrGGGGgrrrrr	40
yyyyyrrrrryyyyyrrrrr	3
rrrrrGGGGgrrrrrGGGGg	40
rrrrryyyyyrrrrryyyyy	3

## Data Availability

The data is all usable and can be provided in its original form.
